# Perioperative and mid-term outcomes of robotic-assisted versus video-assisted minimally invasive esophagectomy for esophageal cancer: a retrospective propensity-matched analysis of 842 patients

**DOI:** 10.3389/fonc.2024.1447393

**Published:** 2024-08-27

**Authors:** Jiang-shan Huang, Jia-fu Zhu, Qi-hong Zhong, Fei-long Guo, Yu-kang Lin, Zhen-yang Zhang, Jiang-bo Lin

**Affiliations:** ^1^ Department of Thoracic Surgery, Fujian Medical University Union Hospital, Fuzhou, China; ^2^ Clinical Research Center for Thoracic Tumors of Fujian Province, Fuzhou, China; ^3^ Key Laboratory of Cardio-Thoracic Surgery, Fujian Medical University, Fujian Province University, Fuzhou, China; ^4^ National Key Clinical Specialty of Thoracic Surgery, Fuzhou, China

**Keywords:** esophageal cancer, invasive minimally esophagectomy, robotic-assisted, video-assisted, mid-term outcomes

## Abstract

**Aim:**

Comparing the safety, effectiveness, and mid-term survival rates of robot-assisted minimally invasive esophagectomy (RAMIE) and video-assisted minimally invasive esophagectomy (VAMIE).

**Methods:**

A total of 842 patients undergoing minimally invasive esophagectomy were analyzed, including 694 patients in VAMIE group and 148 in RAMIE group. PSM analysis was applied to generate matched pairs for further comparison. Operative outcomes, postoperative complications and Mid-term outcomes were compared between all patients in matched groups.

**Results:**

After 1:4 PSM, 148 patients in the RAMIE and 592 patients in the VAMIE. Compared to VAMIE, RAMIE exhibited earlier removal of chest and neck drainage tubes, shorter postoperative hospital stays, and a higher number of lymph node dissections. However, the surgical duration of RAMIE was longer than that of VAMIE. Postoperative complications were no statistically significant between the RAMIE and VAMIE groups. There was no statistically significant difference in the 3-year OS and DFS between the two groups.

**Conclusion:**

Compared to VAMIE, RAMIE emerges as a viable and safe surgical approach and suggests RAMIE as a potential alternative to minimally invasive esophagectomy.

## Background

Esophageal cancer is the seventh most common cancer and the sixth most common cancer in the world (1). About half of the new cases occur in China each year, and the incidence of esophageal cancer in men is higher than that in women. In China, the incidence and mortality of esophageal cancer in men rank the fifth (2). According to the latest evidence guidelines, the main treatment mode for esophageal cancer is surgery-based comprehensive treatment (3). After decades of development of minimally invasive technology, minimally invasive esophagectomy has become the mainstream operation. Video-assisted minimally invasive esophagectomy (VAMIE) can avoid the disadvantages of open surgery such as great trauma, more complications and slow recovery (4). With the continuous development of Da Vinci robot system, robot-assisted minimally invasive esophagectomy (RAMIE) has become another option for the minimally invasive treatment of esophageal cancer (5). In recent years, studies on the short-term and long-term efficacy of RAMIE in the treatment of esophageal cancer have gradually attracted attention. However, as one of the most RAMIE centers in southern China, it is still important to compare the mid-term survival outcomes between RAMIE and VAMIE. The aim of this study is to compare the safety, effectiveness, and mid-term survival rates of RAMIE and VAMIE using propensity score matching (PSM) analysis.

## Methods

### Patient selection

This study reviewed a database of patients who underwent minimally invasive esophagectomy at Fujian Medical University Union Hospital between January 2019 and October 2023. The inclusion criteria: (1) esophageal squamous cell carcinoma was confirmed by preoperative gastroscopy; (2) no distant metastasis was found in preoperative examination and postoperative pathology. Exclusion criteria: (1) previous history of cancer or malignant tumors in other parts of the body; (2) patients with severe lung diseases; (3) esophagectomy with mediastinoscopy; (4) thoracotomy or laparotomy for esophageal cancer resection; (5) Incomplete data. The study recruited 842 patients: 148 in the robotic group and 694 in the laparoscopic group. After 1:4 propensity score matching (PSM), 148 patients in the RAMIE and 592 patients in the VAMIE were finally included in the analysis.

### Surgical techniques and definitions

The patients underwent RAMIE using a da Vinci Surgical System (Intuitive Surgical, Inc., Sunnyvale, CA). All the surgeons completed a comprehensive training course in the training center and obtained the corresponding certification. Before 2019, more than 500 robot-assisted operations (thoracic surgery) have been performed, including more than 50 cases of RAMIE.

The patient underwent standard bilateral lumen endotracheal intubation with general anesthesia and was placed in the left lateral decubitus position. For RAMIE, a 1 cm incision was made in the 6th intercostal space at the mid-axillary line for the observation port. Additional 1 cm incisions were made in the 3rd intercostal space (first robotic arm), 9th intercostal space (second robotic arm), and 4th intercostal space (assistant port). The azygos vein arch was freed and divided, the esophagus was mobilized within the thoracic cavity, and paraesophageal and recurrent laryngeal nerve lymph nodes were dissected. Chest drains were placed as needed. The patient was then repositioned to the supine position, and a 5 cm incision was made along the anterior edge of the left sternocleidomastoid muscle to expose the cervical esophagus. Abdominal procedures were performed laparoscopically, with the observation port located 2 cm left of the umbilicus. The main operating ports were positioned 2 cm below the costal margin at the right midclavicular line and at the intersection of the right midclavicular line and the umbilical level. Assistant ports were placed 2 cm below the costal margin at the left midclavicular line and 2 cm below the xiphoid process. After establishing pneumoperitoneum, the stomach was mobilized along both the greater and lesser curvatures, and the esophageal hiatus was opened. The assistant port under the xiphoid process was extended to 5 cm to create a 3-4 cm wide gastric tube externally. A mechanical anastomosis was performed at the neck, a gastric tube was placed, the incision was sutured, and a neck drain was positioned. For VAMIE, the observation port was placed at the 7th intercostal space along the mid-axillary line, with the main operating ports at the 5th and 9th intercostal spaces along the posterior axillary line, and the assistant port at the 4th intercostal space along the mid-axillary line. The remaining surgical steps were identical to those of RAMIE. Previous articles have described in detail VAMIE and RAMIE for the treatment of esophageal cancer and lymph node dissection (6, 7). The tumor-node-metastasis (TNM) staging was performed using the eighth edition of the AJCC tumor staging system. The definition of postoperative complications has been described in a previous study (8).

### Follow-up evaluation

During the first postoperative year, patients were seen in the outpatient clinic of our hospital every 3 months for physical examination, laboratory evaluation, and chest and abdominal CT or ultrasonography. Subsequently, the patients were followed up every 6 months. According to the specific conditions of the patients, the follow-up evaluation time could be advanced and the frequency could be increased. Follow-up telephone calls were used as a supplement to the outpatient review for patient survival, recurrence, and death.

### Mid-term outcomes

The primary endpoint was the 3-year disease-free survival (DFS), and the secondary endpoints were the 3-year overall survival (OS) and short-term outcomes. DFS was defined as the period from the operation date to the date of first recurrence, death, or the last follow-up. OS was recorded from the date of surgery to the last follow-up date or date of death.

### Statistical methods

To reduce the effect of selection bias, 1:4 propensity score-matching was performed using logistic regression models. Categorical variables are presented as frequencies and percentages, and continuous variables are presented as medians (interquartile ranges). Categorical variables were compared using the chi-square test, Fisher’s exact test, or Mann-Whitney U test, and continuous variables were compared using the t-test or rank-sum test. Logistic regression analysis was used to identify independent risk factors associated with complications. Log-rank tests were used to compare survival curves estimated using Kaplan-Meier methods. All statistical analyses were performed using SPSS v.26.0 for Windows (SPSS Inc., Chicago, Illinois, USA); R (https://www.r-project.org) and GraphPad Prism 9. all reported P values are bidirectional, and P values below 0.05 were considered to indicate a statistically significant difference.

## Results

### Clinicopathologic characteristics

**Figure 1:**
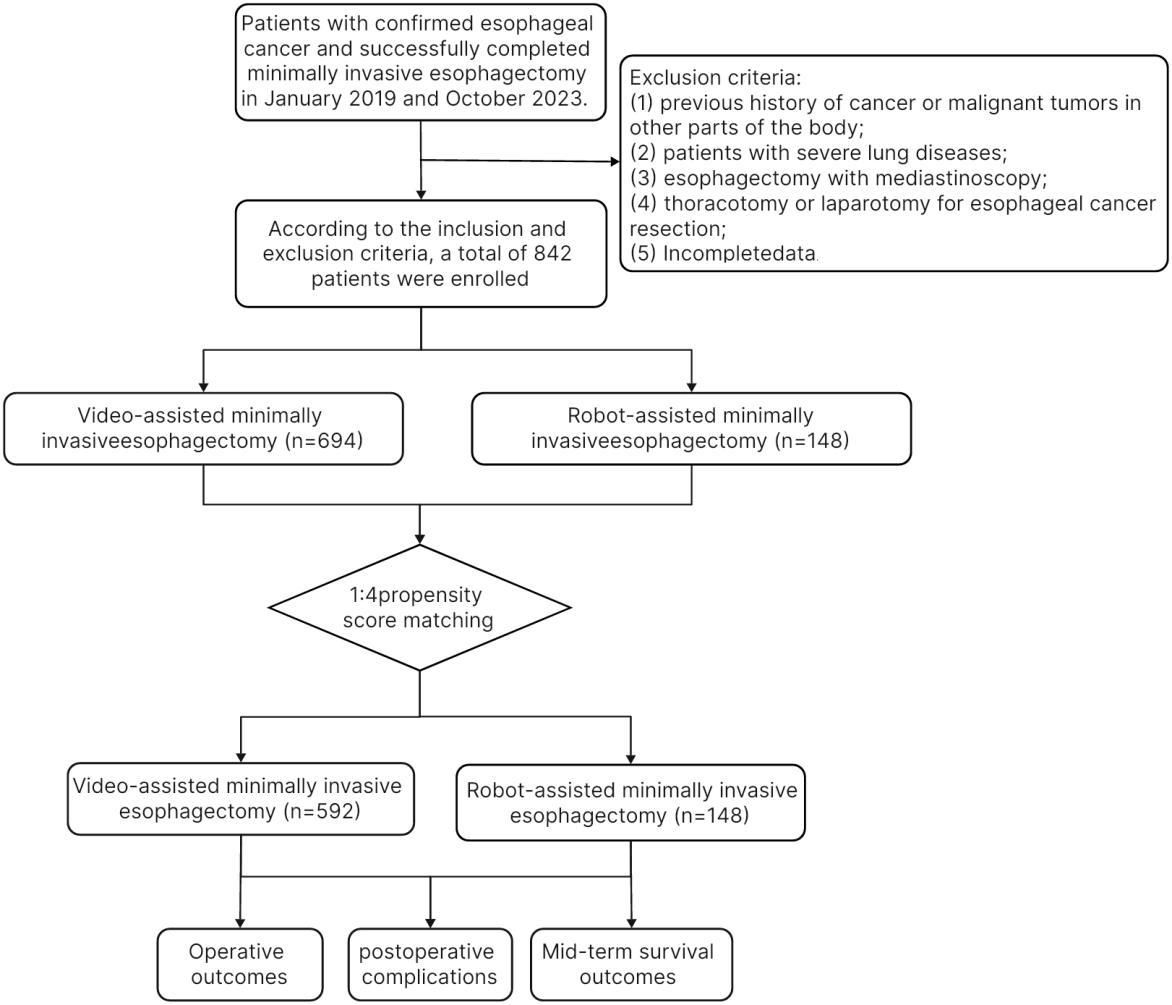
Flowchart of patient enrollment.

**Figure 1** shows the flow chart of patient selection, with 694 patients undergoing video-assisted minimally invasive esophagectomy and 148 patients undergoing robot-assisted minimally invasive esophagectomy. All these patients met the aforementioned inclusion and exclusion criteria. After 1:4 propensity score-matching, 592 patients in the VAMIE group and 148 patients in the RAMIE group were included in the post-matching cohort.

**Table 1** presents the detailed clinicopathological characteristics of patients before (n=842) and after (n=740) PSM in the VAMIE and RAMIE groups. There was a statistically significant difference in age before PSM. After PSM, patient characteristics were balanced between groups (all SMD <0.100).

**Table 1:** Comparison of baseline characteristics of patients in VAMIE and RAMIE.

	unmatched cohort			Marched cohort		
	VAMIE (n=694)	RAMIE (n=148)	p	VAMIE (n=592)	RAMIE (n=148)	p
Sex (Male), n (%)	536 (77.2%)	113 (76.4%)	0.901	450 (76.0%)	113 (76.4%)	0.931
Age (years)	61.0 [56.0;67.0]	64.0 [57.0;68.0]	0.005	63.0 [57.0;67.0]	64.0 [57.0;68.0]	0.197
Smoking history (+), n (%)	389 (56.1%)	76 (51.4%)	0.341	317 (53.5%)	76 (51.4%)	0.699
Weight (kg)	60.0 [52.5;67.0]	60.0 [55.0;66.0]	0.296	60.0 [53.0;67.2]	60.0 [55.0;66.0]	0.561
BMI (kg/m^2^)	21.8 [20.0;23.8]	21.8 [20.5;23.8]	0.393	22.0 [20.3;24.0]	21.8 [20.5;23.8]	0.950
MVV (L)	98.7 [82.4;111]	96.9 [83.3;110]	0.544	98.2 [82.0;111]	96.9 [83.3;110]	0.772
CEA	2.30 [1.50;3.50]	2.40 [1.50;3.40]	0.679	2.30 [1.50;3.32]	2.40 [1.50;3.40]	0.428
Alcohol consumption (+), n (%)	221 (31.8%)	41 (27.7%)	0.373	169 (28.5%)	41 (27.7%)	0.919
Hypertension (+), n (%)	133 (19.2%)	32 (21.6%)	0.569	121 (20.4%)	32 (21.6%)	0.838
Diabetes (+), n (%)	61 (8.79%)	11 (7.43%)	0.708	46 (7.77%)	11 (7.43%)	0.890
CHD (+), n (%)	38 (5.48%)	11 (7.43%)	0.465	35 (5.91%)	11 (7.43%)	0.621
Neoadjuvant (+), n (%)	325 (46.8%)	74 (50.0%)	0.542	280 (47.3%)	74 (50.0%)	0.619
Location of tumor:			0.863			0.789
upper	52 (7.49%)	11 (7.43%)		44 (7.43%)	11 (7.43%)	
middle	335 (48.3%)	68 (45.9%)		290 (49.0%)	68 (45.9%)	
Lower	307 (44.2%)	69 (46.6%)		258 (43.6%)	69 (46.6%)	
Pathological staging			0.331			0.358
0	45 (6.49%)	15 (10.1%)		44 (7.43%)	15 (10.1%)	
1	199 (28.7%)	40 (27.0%)		176 (29.7%)	40 (27.0%)	
2	184 (26.6%)	31 (20.9%)		158 (26.7%)	31 (20.9%)	
3	242 (34.9%)	58 (39.2%)		195 (32.9%)	58 (39.2%)	
4	23 (3.32%)	4 (2.70%)		19 (3.21%)	4 (2.70%)	

BMI, Body Mass Index; MVV, maximum ventilatory volume; CHD, chronic heart disease.

### Surgical results and short-term outcomes

The mean operative time was significantly longer in RAMIE group (345 minutes) than in VAMIE group (313 minutes) (P <0.001). The mean total of lymph node dissection in RAMIE group was significantly higher than that in VAMIE group (P <0.001). Similarly, RAMIE had earlier chest (P <0.001) and neck (P <0.001) drainage tube removal and shorter postoperative hospital stay than VAMIE(P=0.003). However, there was no significant difference in the amount of intraoperative blood transfusion (P=0.846) and the number of patients needing blood transfusion after operation (P=0.793) between the two groups. Postoperative pathology showed that there was no significant difference in vascular and nerve invasion between the two groups. (Details are provided in **Table 2**).

**Table 2:** Comparison of Perioperative Data between Matched VAMIE and RAMIE Groups.

	VAMIE (n=592)	RAMIE (n=148)	p
Surgical Duration	313 [280;360]	345 [306;390]	<0.001
Position of anastomosis:			<0.001
Neck	571 (96.5%)	131 (88.5%)	
Intrathoracic	21 (3.55%)	17 (11.5%)	
Intraoperative Blood Loss (ml)	100 [50.0;100]	100 [50.0;100]	0.846
Lymph Node Dissection Count	33.0 [25.0;42.0]	35.5 [28.0;46.2]	0.002
RLN Lymph Nodes	3.00 [3.00;4.00]	3.00 [2.0;4.00]	0.047
Mediastinal Lymph Nodes	21.5 [13.0;30.0]	24.0 [17.0;35.2]	0.005
Abdominal Lymph Nodes	8.00 [7.00;9.00]	7.00 [5.00;11.0]	0.135
Vascular invasion (+), n (%)	106 (17.9%)	29 (19.6%)	0.721
Nerve infiltration (+), n (%)	159 (26.9%)	41 (27.7%)	0.918
Thoracic Extubation Time	8.00 [7.00;11.2]	8.00 [6.00;9.00]	<0.001
Neck Extubation Time	6.00 [4.00;8.00]	5.00 [4.00;6.00]	<0.001
Hospitalization days (day)	10.0 [8.00;14.0]	9.00 [8.00;13.0]	0.003
Postoperative Transfused Patients, n (%)	18 (3.04%)	5 (3.38%)	0.793

RLN, Recurrent Laryngeal Nerve.

**Table 3** presents the postoperative complications. There were no significant differences between RAMIE group and VAMIE group in postoperative anastomotic leakage, postoperative pneumonia, hepatic and renal dysfunction, pleural effusion, chylothorax, arrhythmia and hoarseness. Second, we aimed to identify the associated independent risk factors for postoperative complications. In univariate regression analysis, CEA (P=0.07), hepatorenal Dysfunction (P=0.038), tumor location (P <0.001), and time to neck (P <0.001) and chest extubation (P <0.001) were associated with postoperative complications, but not with surgical procedure. To further explore, multivariate regression analysis showed that chest extubation time, tumor location and history of alcohol consumption were independent risk factors (**Table 4**).

**Table 3:** Comparison of postoperative complications between VAMIE and RAMIE.

	VAMIE (n=592)	RAMIE (n=148)	P
EGAL(+), n (%)	72 (12.2%)	23 (15.5%)	0.336
Pulmonary Infection (+), n (%)	194 (32.8%)	38 (25.7%)	0.118
Reoperation (+), n (%)	6 (1.02%)	1 (0.68%)	0.927
Hepatorenal Dysfunction (+), n (%)	68 (11.5%)	13 (8.78%)	0.427
Pleural Effusion (+), n (%)	49 (8.28%)	16 (10.8%)	0.417
Chylothorax (+), n (%)	8 (1.35%)	1 (0.68%)	0.696
Arrhythmia (+), n (%)	41 (6.93%)	9 (6.08%)	0.855
Hoarseness (+), n (%)	46 (9.29%)	14 (11.2%)	0.635

EGAL, esophagogastric anastomotic leakage.

**Table 4:** Uni- and multivariate analysis of risk factors for postoperative complications in the propensity score-matched cohort.

Variables	Univariate analysis		Multivariate analysis	
	CI	P	CI	P
Surgical approach	0.773(0.538-1.109)	0.163	0.937(0.631-1.388)	0.746
Neck Extubation Time	1.124(1.08-1.174)	<0.001	1.048(0.996-1.105)	0.078
Thoracic Extubation Time	1.162(1.117-1.212)	<0.001	1.141(1.086-1.203)	<0.001
Lymph Node Dissection Count	1.002(0.991-1.012)	0.757		
pTNM stage	1.217(0.903-1.641)	0.197		
Intraoperative Blood Loss	0.999(0.996-1.001)	0.198		
Surgical Duration	0.999(0.997-1.001)	0.531		
Location of tumor	0.576(0.429-0.771)	<0.001	0.499(0.36-0.688)	<0.001
Neoadjuvant	0.862(0.646-1.151)	0.315		
CHD	1.15(0.632-2.111)	0.647		
Diabetes	1.25(0.727-2.172)	0.421		
Hypertension	0.963(0.674-1.375)	0.834		
Alcohol consumption	1.406(1.02-1.943)	0.038	1.619(1.107-2.379)	0.013
CEA	1.066(1.003-1.152)	0.07	1.04(0.974-1.124)	0.279
MVV	1.005(0.997-1.012)	0.24		
BMI	1.008(0.959-1.059)	0.763		
weight	1.007(0.993-1.022)	0.338		
smokeing	1.334(0.999-1.783)	0.051	1.12(0.795-1.577)	0.516
age	1.015(0.996-1.036)	0.129	1.011(0.99-1.034)	0.309
sex	0.959(0.684-1.345)	0.807		

BMI, Body Mass Index; MVV, maximum ventilatory volume; CHD, chronic heart disease.

### Mid-term outcomes

**Figure 2:**
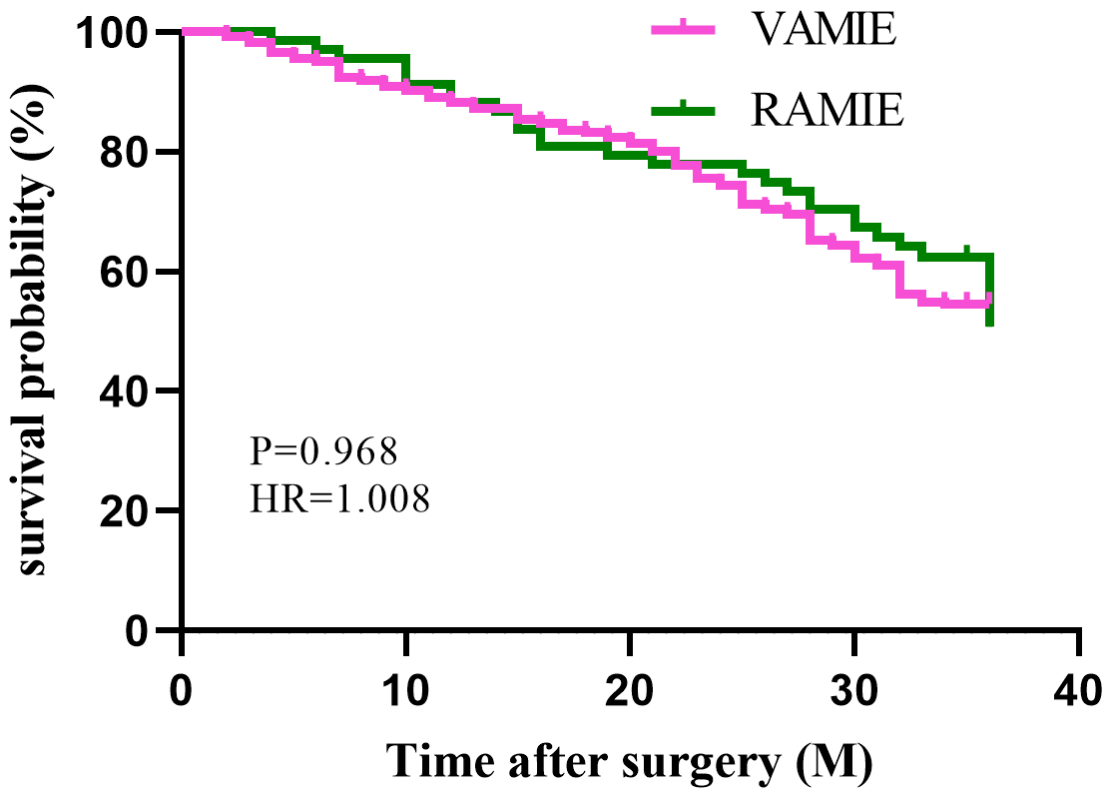
Kaplan−Meier curves comparing overall survival between patients in the RAMIE and VAMIE.

**Figure 3:**
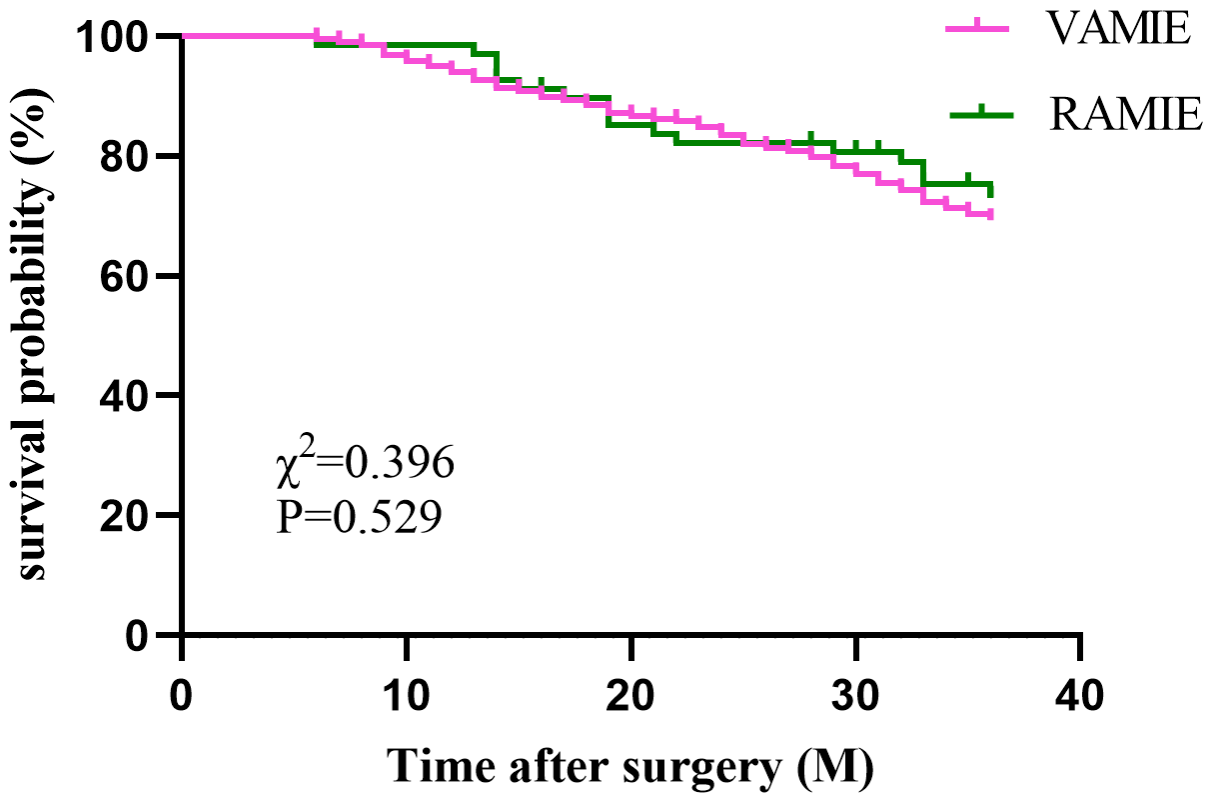
Kaplan−Meier curves comparing disease-free survival between patients in the RAMIE and VAMIE.

In the post-PSM cohort, a total of 450 patients who had been followed up for 36 months were included for survival analysis. The 3-year OS rates were 75% in the RAMIE group and 70.9% in the VAMIE group (**Figure 2**), with no significant difference between the two groups (P=0.529). Similarly, the 3-year DFS rates in the RAMIE and VAMIE groups were 54.4% and 56.1%, respectively (**Figure 3**), with no statistically significant difference (P=0.968).

## Discussion

In 2003, Horgan S et al. first reported RAMIE (9). Over the following decade, many medical centers adopted RAMIE (5, 10). Previous studies indicated that RAMIE had better short-term outcomes compared to VAMIE (11). However, few studies have compared the mid- to long-term prognoses of RAMIE and VAMIE. Our study, with a large sample size analyzing data from nearly 900 patients, is one of the largest comparative analyses to date. Unlike other studies, we not only provide short-term outcomes but also compare the 3-year OS and DFS rates, offering a mid-term survival analysis. The use of 1:4 propensity score matching (PSM) further enhances the statistical power and reliability of our findings. Our center is one of the largest thoracic surgery centers in Southern China, representing the characteristics of the Southern Chinese population. Therefore, conducting this study is essential. Our results demonstrate that with advancements in robotic technology and surgical techniques, RAMIE can be a feasible alternative with outcomes similar to or better than VAMIE. Thus, our study supports the widespread adoption of RAMIE in clinical practice.

Our research findings indicate that the RAMIE procedure, while longer in duration compared to VAMIE surgery, we consider this discrepancy to be attributable to the additional time required for the setup of the system. Unfortunately, our case system did not record the installation time, thus this study is unable to conclusively establish a difference in operative time between the two methods. Our lead surgeons at our center had each completed over 50 cases of da Vinci esophageal cancer radical surgery by 2019 and obtained certification qualifications. RUURDA’s meta-analysis included 16 studies with a total of 300 cases of RAMIE. The results showed that the majority of thoracic centers identified 20 cases as the threshold for completing the learning curve for RAMIE (12). SARKARIA et al. analyzed the learning curve of 100 cases of RAMIE and concluded that, due to the proficiency of the majority of thoracic surgeons in open and laparoscopic surgeries, after 20 to 30 cases of experience, surgeons can proficiently master the operative techniques of RAMIE (13). Hence, it can be considered that the lead surgeons at our center have fully mastered the operative techniques of RAMIE. “Babic B suggests that there is no statistically significant difference in the thoracic time between the two procedures (14), while Weksler B proposes that there is no apparent disparity in the actual operating time between the two procedures (15).

We found a significantly higher number of lymph node dissections in the RAMIE group compared to the VAMIE group, which is consistent with the findings of Ekeke CN (16). This may be attributed to the advantage of RAMIE in clearing mediastinal lymph nodes, particularly in the narrow space of bilateral recurrent laryngeal nerve paratracheal lymph node dissection and protection of the recurrent laryngeal nerve (17). Additionally, there was no statistically significant difference between the two groups in postoperative complications such as hoarseness, indicating that the da Vinci system enables the clearance of more lymph nodes while adequately protecting the recurrent laryngeal nerve. Benefiting from the flexible robotic arms, a three-dimensional surgical field of view, and stable mechanical arms, RAMIE surgery enables precise operations within confined spaces, resulting in reduced trauma. This may contribute to the shorter extubation time (both neck and chest) and length of hospital stay observed in the da Vinci group. However, specific reasons still require further exploration.

In this study, we observed that postoperative complications, including pulmonary infection, anastomotic fistula, hoarseness, and chylothorax, did not significantly differ between the RAMIE and VAMIE groups. Through multivariate regression analysis of postoperative complications, we identified tumor location, history of alcohol consumption, and time of chest tube removal as independent risk factors, warranting further exploration. This avenue represents one of the focal points for our subsequent investigations. Betzler J et al. conducted a comparative analysis of perioperative outcomes between RAMIE and VAMIE, revealing lower incidences of postoperative complications, anastomotic fistula, and ICU stay among RAMIE patients (18). Integrating these findings with our own, RAMIE emerges as a safe surgical modality with notable advantages in lymph node dissection and length of hospital stay. Our results indicate that RAMIE demonstrates similar OS and DFS compared to VAMIE at 3 years postoperatively. A study from the National Cancer Database (NCDB) similarly suggests that RAMIE and VAMIE exhibit comparable survival rates compared to open thoracotomy esophagectomy, with no significant differences observed in OS (19), aligning closely with our findings.

## Limitations

This study has several limitations. Firstly, despite the utilization of PSM analysis, potential selection bias cannot be entirely excluded. Secondly, although a 3-year follow-up period was conducted, a longer follow-up duration is required to observe long-term effects. Thirdly, the retrospective nature of the individual review data in this study is derived from our single center, which may not accurately assess the advantages of RAMIE and VAMIE. Therefore, we are preparing to conduct a prospective multicenter clinical study to compare the roles of these two methods in the treatment of esophageal cancer.

## Conclusion

Compared to VAMIE, RAMIE emerges as a viable and safe surgical approach, exhibiting certain advantages in hospitalization duration, extubation time, and lymph node dissection. The mid-term survival outcomes between the two procedures are comparable, suggesting RAMIE as a potential alternative to minimally invasive esophagectomy.

## Data Availability

The original contributions presented in the study are included in the article/**Supplementary Material**. Further inquiries can be directed to the corresponding authors.
