# Supplementary figures and images for: Perioperative and mid-term outcomes of robotic-assisted versus video-assisted minimally invasive esophagectomy for esophageal cancer: a retrospective propensity-matched analysis of 842 patients
Source: Front Oncol. 2024 Aug 27;14:1447393. doi: 10.3389/fonc.2024.1447393 (PMC11385285; doi:10.3389/fonc.2024.1447393)

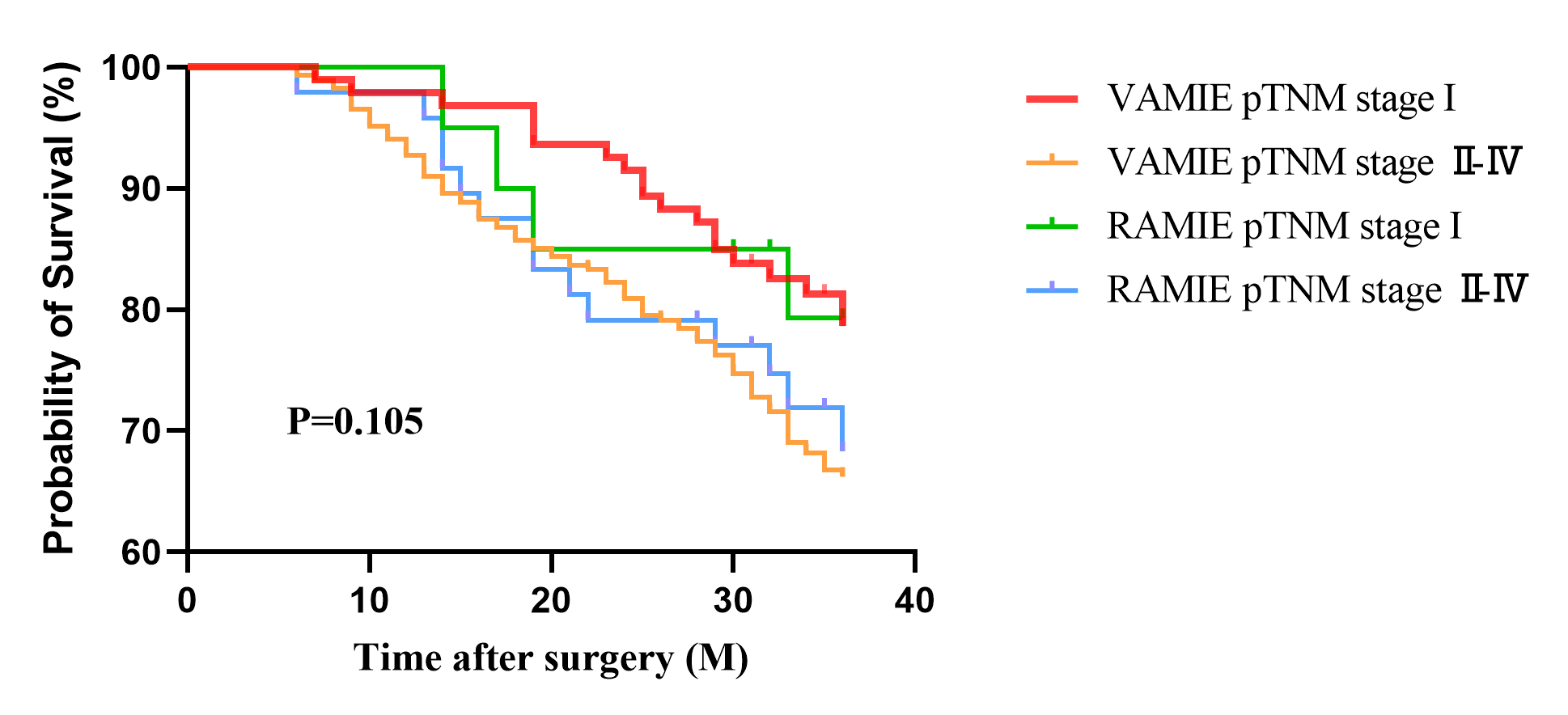

Supplement: Supplementary file 2 [file Image1.tif]

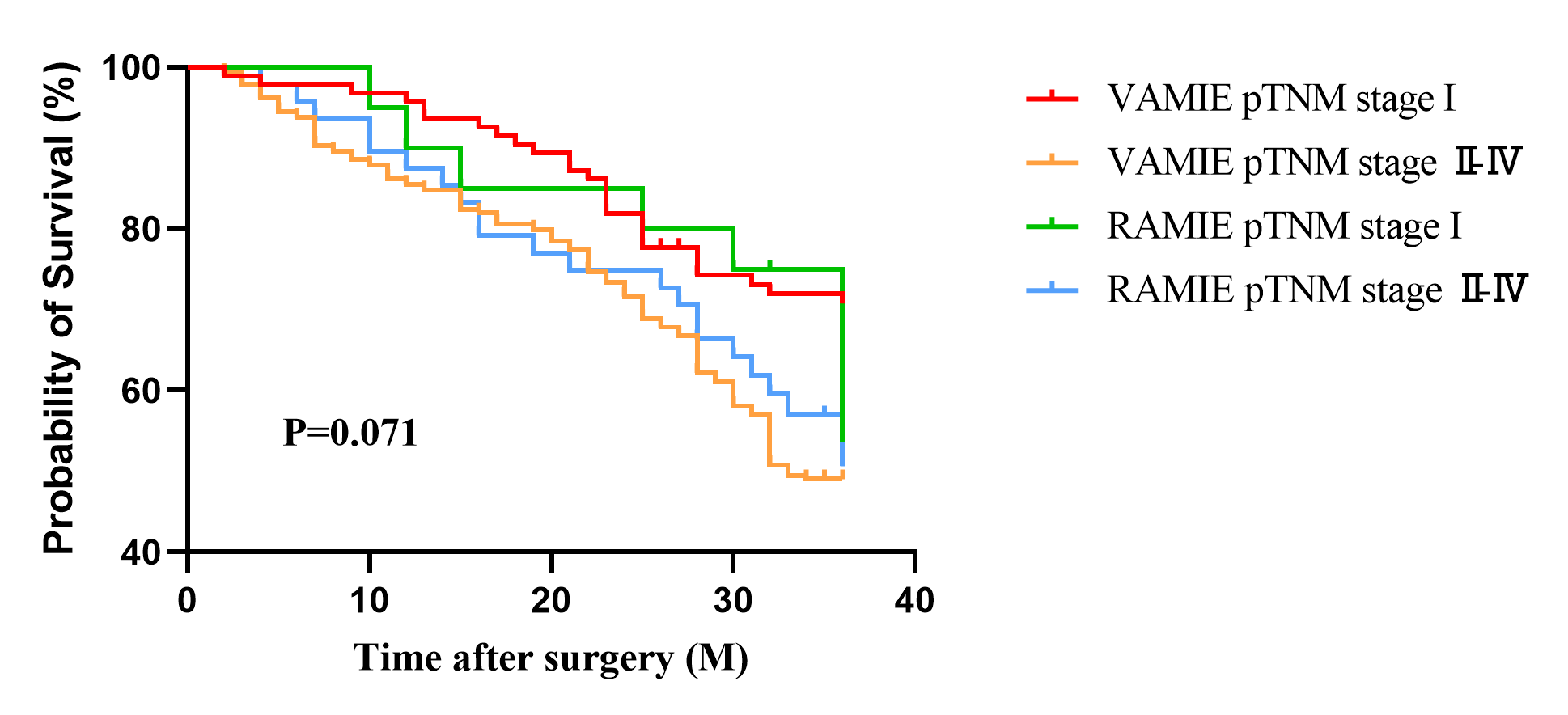

Supplement: Supplementary file 3 [file Image2.tif]
