# Supplementary material for: Perioperative and mid-term outcomes of robotic-assisted versus video-assisted minimally invasive esophagectomy for esophageal cancer: a retrospective propensity-matched analysis of 842 patients
Source: Front Oncol. 2024 Aug 27;14:1447393. doi: 10.3389/fonc.2024.1447393 (PMC11385285; doi:10.3389/fonc.2024.1447393)
Supplement: Supplementary file 4 [file Table1.docx]

| Author | Year | included studies | included patients | | outcomes |
| --- | --- | --- | --- | --- | --- |
|  |  |  | MIE（or OE） | RAMIE |  |
| Zhang Y et al. | 2023 | 18 | 1514 | 1418 | RAMIE shows more lymph node dissection; 3-year overall survival is similar, but disease-free survival is better; no difference in perioperative acute complications (except pneumonia) between the two groups |
| Perry R et al. | 2024 | 18 | 13491 | 4696 | RAMIE has a longer operation time; lower pulmonary complications, no difference in other complications; faster recovery and better quality of life |
| Esagian SM et al. | 2022 | 10 | 1304 | 674 | RAMIE results in fewer cases of pneumonia, wound infections, and atrial fibrillation, with shorter hospital stays and longer operation times. There is no difference in short-term oncological outcomes. |
| Zhou J et al. | 2022 | 20 | 5275 | 974 | RAMIE results in fewer pulmonary complications, shorter hospital stays, and more extensive lymph node dissection |
| Huang Y et al. | 2021 | 15 | 1514 | 1418 | RAMIE has a longer operation time, fewer pulmonary complications, and no difference in other complications. |
| Mederos MA et al. | 2021 | 21 | 6423 | 2932 | RAMIE has fewer complications and less blood loss. |
| Angeramo CA et al. | 2021 | 20 | 5275 | 974 | Patients undergoing RAMIE have less intraoperative blood loss, lower postoperative pneumonia incidence and overall morbidity, and a higher R0 resection rate. |
| Manigrasso M et al. | 2021 | 18 | 1513 | 1418 | Compared to MIE, RAMIE has advantages in intraoperative blood loss, postoperative pulmonary complications, and lymph node dissection, but the operation time is longer |
| Zheng C et al. | 2021 | 14 | 1452 | 1435 | RAMIE shows superiority in reducing postoperative pneumonia and vocal cord paralysis |
| Jin D et al. | 2019 | 19 | 1840 | 1642 | Compared to MIE, RAMIE has advantages in terms of intraoperative blood loss, postoperative pneumonia incidence, and hospital stay duration, but the operation time is longer |

Reference

1. Zhang Y, Dong D, Cao Y, Huang M, Li J, Zhang J, Lin J, Sarkaria IS, Toni L, David R, He J, Li H. Robotic Versus Conventional Minimally Invasive Esophagectomy for Esophageal Cancer: A Meta-analysis. Ann Surg. 2023 Jul 1;278(1):39-50.
2. Perry R, Barbosa JP, Perry I, Barbosa J. Short-term outcomes of robot-assisted versus conventional minimally invasive esophagectomy for esophageal cancer: a systematic review and meta-analysis of 18,187 patients. J Robot Surg. 2024 Mar 16;18(1):125.
3. Esagian SM, Ziogas IA, Skarentzos K, Katsaros I, Tsoulfas G, Molena D, Karamouzis MV, Rouvelas I, Nilsson M, Schizas D. Robot-Assisted Minimally Invasive Esophagectomy versus Open Esophagectomy for Esophageal Cancer: A Systematic Review and Meta-Analysis. Cancers (Basel). 2022 Jun 29;14(13):3177.
4. Zhou J, Xu J, Chen L, Hu J, Shu Y. McKeown esophagectomy: robot-assisted versus conventional minimally invasive technique-systematic review and meta-analysis. Dis Esophagus. 2022 Oct 14;35(10):doac011.
5. Huang Y, Zhao YL, Song JD. Early outcomes with robot-assisted vs. minimally invasive esophagectomy for esophageal cancer: a systematic review and meta-analysis of matched studies. Eur Rev Med Pharmacol Sci. 2021 Dec;25(24):7887-7897.
6. Mederos MA, de Virgilio MJ, Shenoy R, Ye L, Toste PA, Mak SS, Booth MS, Begashaw MM, Wilson M, Gunnar W, Shekelle PG, Maggard-Gibbons M, Girgis MD. Comparison of Clinical Outcomes of Robot-Assisted, Video-Assisted, and Open Esophagectomy for Esophageal Cancer: A Systematic Review and Meta-analysis. JAMA Netw Open. 2021 Nov 1;4(11):e2129228.
7. Angeramo CA, Bras Harriott C, Casas MA, Schlottmann F. Minimally invasive Ivor Lewis esophagectomy: Robot-assisted versus laparoscopic-thoracoscopic technique. Systematic review and meta-analysis. Surgery. 2021 Dec;170(6):1692-1701.
8. Manigrasso M, Vertaldi S, Marello A, Antoniou SA, Francis NK, De Palma GD, Milone M. Robotic Esophagectomy. A Systematic Review with Meta-Analysis of Clinical Outcomes. J Pers Med. 2021 Jul 6;11(7):640.
9. Zheng C, Li XK, Zhang C, Zhou H, Ji SG, Zhong JH, Xu Y, Cong ZZ, Wang GM, Wu WJ, Shen Y. Comparison of short-term clinical outcomes between robot-assisted minimally invasive esophagectomy and video-assisted minimally invasive esophagectomy: a systematic review and meta-analysis. J Thorac Dis. 2021 Feb;13(2):708-719.
10. Jin D, Yao L, Yu J, Liu R, Guo T, Yang K, Gou Y. Robotic-assisted minimally invasive esophagectomy versus the conventional minimally invasive one: A meta-analysis and systematic review. Int J Med Robot. 2019 Jun;15(3):e1988.
